# Detection of municipalities at-risk of Lyme disease using passive surveillance of *Ixodes scapularis* as an early signal: A province-specific indicator in Canada

**DOI:** 10.1371/journal.pone.0212637

**Published:** 2019-02-19

**Authors:** Salima Gasmi, Nicholas H. Ogden, Marion Ripoche, Patrick A. Leighton, Robbin L. Lindsay, Mark P. Nelder, Erin Rees, Catherine Bouchard, Linda Vrbova, Richard Rusk, Curtis Russell, Yann Pelcat, Samir Mechai, Serge-Olivier Kotchi, Jules K. Koffi

**Affiliations:** 1 Policy Integration and Zoonoses Division, Centre for Food-borne, Environmental and Zoonotic Infectious Diseases, Public Health Agency of Canada, Saint-Hyacinthe, Québec, Canada; 2 Groupe de recherche en épidémiologie des zoonoses et santé publique, Faculty of Veterinary Medicine, University of Montréal, Saint-Hyacinthe, Québec, Canada; 3 Public Health Risk Sciences Division, National Microbiology Laboratory, Public Health Agency of Canada, Saint-Hyacinthe, Québec, Canada; 4 Department of Pathology and Microbiology, Faculty of Veterinary Medicine, University of Montréal, Saint-Hyacinthe, Québec, Canada; 5 Zoonotic Diseases and Special Pathogens Division, National Microbiology Laboratory, Public Health Agency of Canada, Winnipeg, Manitoba, Canada; 6 Enteric, Zoonotic and Vector-Borne Diseases, Communicable Diseases, Emergency Preparedness and Response, Public Health Ontario, Toronto, Ontario, Canada; 7 Centre for Food-borne, Environmental & Zoonotic Infectious Diseases, Public Health Agency of Canada, Toronto, Ontario, Canada; 8 Active Living, Indigenous Relations, Population & Public Health Division, Communicable Disease Control Branch, Manitoba Health, Seniors & Active Living, Winnipeg, Manitoba, Canada; University of Kentucky College of Medicine, UNITED STATES

## Abstract

Lyme disease, the most commonly reported vector-borne disease in North America, is caused by the spirochete *Borrelia burgdorferi* sensu stricto, which is transmitted by *Ixodes scapularis* in eastern Canada and *Ixodes pacificus* in western Canada. Recently, the northward range expansion of *I*. *scapularis* ticks, in south-eastern Canada, has resulted in a dramatic increase in the incidence of human Lyme disease. Detecting emerging areas of Lyme disease risk allows public health to target disease prevention efforts. We analysed passive tick surveillance data from Ontario and Manitoba to i) assess the relationship between the total numbers of *I*. *scapularis* submissions in passive surveillance from humans, and the number of human Lyme disease cases, and ii) develop province-specific acarological indicators of risk that can be used to generate surveillance-based risk maps. We also assessed associations between numbers of nymphal *I*. *scapularis* tick submissions only and Lyme disease case incidence. Using General Estimating Equation regression, the relationship between *I*. *scapularis* submissions (total numbers and numbers of nymphs only) in each census sub-division (CSD) and the number of reported Lyme disease cases was positively correlated and highly significant in the two provinces (*P* ≤ 0.001). The numbers of *I*. *scapularis* submissions over five years discriminated CSDs with ≥ 3 Lyme disease cases from those with < 3 cases with high accuracy when using total numbers of tick submission (Receiver Operating Characteristics area under the curve [AUC] = 0.89) and moderate accuracy (AUC = 0.78) when using nymphal tick submissions only. In Ontario the optimal cut-off point was a total 12 tick submissions from a CSD over five years (Sensitivity = 0.82, Specificity = 0.84), while in Manitoba the cut-off point was five ticks (Sensitivity = 0.71, Specificity = 0.79) suggesting regional variability of the risk of acquiring Lyme disease from an *I*. *scapularis* bite. The performances of the acarological indicators developed in this study for Ontario and Manitoba support the ability of passive tick surveillance to provide an early signal of the existence Lyme disease risk areas in regions where ticks and the pathogens they transmit are expanding their range.

## Introduction

Lyme disease, a multisystemic infection caused by the spirochetal bacterium *Borrelia burgdorferi* sensu stricto (*B*. *burgdorferi*), is the most commonly reported vector-borne disease in North America. In central and eastern part of the continent, the main vector of *B*. *burgdorferi* is the blacklegged tick, *Ixodes scapularis*. *Ixodes scapularis* can also transmit other pathogens of public health concern such as *Anaplasma phagocytophilum*, *Babesia microti*, *Borrelia miyamotoi*, deer tick virus (Powassan virus lineage II) and *Ehrlichia muris-*like bacteria [1]. Driven in part by a warming climate, the geographic range of *I*. *scapularis* and the diseases it transmits has expanded northwards into Canada, from the US [2]. Continued northwards range expansion of the tick is expected as environmental suitability increases [3]. At a local scale, this geographic expansion may, however, be patchy and discontinuous [4] as a result of other environmental drivers, such as humidity [5], vegetation type [3, 6, 7], forest fragmentation [8, 9], biodiversity [10] and density of *B*. *burgdorferi* reservoirs [7, 11], as well as by stochastic fade-out of invading tick populations [12]. Blacklegged tick population density and prevalence of *B*. *burgdorferi* infection in ticks are also temporally dynamic, with both increasing over the years after both tick and bacterium become established for the first time [13]. In 2009, Lyme disease became nationally notifiable in Canada, and since then the number of locations where blacklegged ticks have been found has increased exponentially in south-eastern Canada [14–16]. The rapid expansion of the geographic scope of Lyme disease risk areas (i.e. areas where reproducing populations of *I*. *scapularis* occur and are maintaining *B*. *burgdorferi* transmission cycles) makes the detection of areas where the risk of acquiring Lyme disease occurs a priority for public health authorities. Detection of these areas allows targeted preventive and control measures for the public, guides general practitioners in their diagnosis of Lyme disease infections in patients with history of exposure to risk areas, and helps inform reporting of cases in public health surveillance systems, thus contributing to efforts to minimise the impact of emerging Lyme disease.

The Canadian Lyme disease surveillance program consists of the collection of data on human cases and environmental risk (i.e. where *B*. *burgdorferi*-transmitting blacklegged ticks occur). The Provincial public health departments or provincial laboratories investigate reports of human Lyme disease cases and then submit data on these cases via the Canadian Notifiable Disease Surveillance System (CNDSS) and the Lyme Disease Enhanced Surveillance (LDES) system of the Public Health Agency of Canada [14]. The blacklegged tick component of the Lyme disease surveillance program consists of two parts: passive and active field tick surveillance. In some provinces, passive tick surveillance involves voluntary medical and veterinary clinics submitting ticks collected on humans and animals, respectively [17], while in other provinces, ticks can be submitted by the general public through local public health authorities. Active field tick surveillance consists of the collection of questing blacklegged ticks from the environment by drag sampling or through the capture and examination of tick hosts [18]. In Lyme disease human surveillance, diagnosis and reporting of cases in emerging risk areas may be limited by many factors, including Lyme disease awareness campaigns, practices of front-line health professionals, the competing priorities of the provincial public health systems, as well as differences in methods of ascertainment and verification of cases. Active field surveillance is the gold standard method for detection of tick populations and Lyme disease risk in the environment. It is, however, resource-heavy, and it is impractical to consider conducting active surveillance in every possible area of suitable habitat in Canada. Consequently, active field surveillance is often conducted only in areas where established tick populations are suspected based on signals from other methods of surveillance [19] or in the course of research activities [20].

It has been shown that levels of environmental risk (defined as the density of host-seeking infected blacklegged ticks or infected nymphal blacklegged ticks estimated in active tick surveillance) are correlated with the incidence of human Lyme disease where tick populations and tick-borne pathogen cycles have become established [21–23]. Domestic pets, particularly dogs, are particularly good at acquiring ticks in the environment [24]. Adventitious ticks can be detected by passive surveillance, producing false-positive locations for reproducing tick populations, and this is particularly likely when using ticks collected from dogs [17]. Nevertheless, by careful analysis, passive tick surveillance data can be used to identify regions where *I*. *scapularis* tick populations may be becoming established [25]. Recently, Ripoche et al. (2018) investigated the relationship between the Lyme disease risk signals provided by reported Lyme disease human cases and passive and active blacklegged tick surveillance in Québec [26]. Evidence of tick populations provided by both active and passive surveillance were positively associated with human Lyme disease cases; however, the relationship between passive *I*. *scapularis* surveillance and reported Lyme disease human cases was the strongest. Moreover, this study demonstrated that the best indicator of municipalities with at least three locally acquired human Lyme disease cases was the cumulative number of *I*. *scapularis* tick submissions from human origin over a five-year period. An incidence of three Lyme disease cases over five years has been considered as being consistent with the presence of *B*. *burgdorferi*-transmitting *I*. *scapularis* populations in the province of Quebec in Canada [27], and in the past, the presence of two Lyme disease cases was considered as indicating endemicity in the US (https://wwwn.cdc.gov/nndss/conditions/lyme-disease/case-definition/2008/). In Quebec, a cut-off point of 11 *I*. *scapularis* tick submissions performed well at discriminating municipalities in which incidence was ≥ or < 3 cases over five years[26]. This study showed that as long as of human population density (and numbers of participating medical clinics) is not too low or high (in densely populated cities numbers of adventitious blacklegged tick submissions can be above cut-off point levels in the absence of reproducing tick populations), passive surveillance has the potential to be timely in detecting regions where tick populations are becoming established, and has wider geographic coverage than active tick surveillance. Consequently, in some provinces, evidence of reproducing tick populations from passive surveillance are now included in producing risk maps [28, 29]. Here we explored the potential for this approach to analysis of passive tick surveillance data to be adapted to the provinces of Ontario and Manitoba where *I*. *scapularis*, and associated pathogens are emerging. From 2009 through 2015, the incidence of locally acquired human Lyme disease cases rose from 0.5 to 2.7 and from 0.3 to 2.3 cases per 100,000 population in Ontario and Manitoba, respectively [14]. These findings were consistent with our knowledge of the current range expansion of *I*. *scapularis* in these two provinces [30].

## Methods

### Data collected through passive tick surveillance and human Lyme disease surveillance

Lyme disease became nationally notifiable in Canada in 2009, and Lyme disease cases are reported through the CNDSS as well as the LDES system. The CNDSS collects demographic data (age and sex), the date of episode onset and the classification the cases as probable or confirmed. The LDES system also captures data on probable exposure location, clinical manifestations and results of laboratory testing for evidence of infection with *B*. *burgdorferi*. The provinces of Manitoba and Ontario have participated in the LDES system since 2009. We used probable and confirmed cases reported by these provinces from 2009 through 2015 according to the national notifiable Lyme disease definition of 2009 [31]. A confirmed case is a patient with clinical evidence of illness with laboratory confirmation (isolation of *B*. *burgdorferi* or detection of *B*. *burgdorferi* DNA by polymerase chain reaction [PCR] using an appropriate clinical specimen) or with clinical evidence of illness with a history of residence in, or visit to, Lyme disease risk area (i.e. where there is evidence that reproducing tick vector populations are present) and with laboratory evidence of infection, i.e., a positive two-tier (ELISA and immunoblot criteria) serological test result interpreted according to published criteria [32]. A probable case is i) a patient with clinical evidence of illness without a history of residence in, or visit to, an endemic area but with laboratory evidence of infection (positive serological tests using the two-tier algorithm of an ELISA followed by an immunoblot assay, when indicated) or ii) a patient with clinician-observed erythema migrans without laboratory evidence but with history of residence in, or visit to, a Lyme disease risk area.

Information on the residence in, or visit to, a Lyme disease risk area of the Lyme disease cases is ascertained by the public health authority of each province during the case investigation. The location of Lyme disease risk areas is identified mostly by active tick surveillance in Canada, and this information is available on provincial and national public health websites to assist public health practitioners to classify cases [30].

Blacklegged tick data for this study were gathered as part of the Ontario and Manitoba passive tick surveillance programs. In these surveillance systems, ticks found on humans are submitted by participating medical clinics, or by the public directly, to the provincial laboratories or through regional health units. Using standard taxonomic keys, ticks are identified to species, sex and stage (larvae, nymphs, and adults). Ticks identified as *I*. *scapularis* by the provincial laboratories are sent to the National Microbiology Laboratory of the Public Health Agency of Canada for PCR testing for *B*. *burgdorferi* [33].

For this study, the spatial resolution for the likely location of acquisition of *I*. *scapularis* ticks and the locality where Lyme disease infection was acquired was the Census Subdivision (CSD). This geographic unit is defined by Statistics Canada as: ‘an area that is a municipality or an area that is deemed to be equivalent to a municipality for statistical reporting purposes (e.g., as an Indian reserve or an unorganized territory). Municipal status is defined by laws in effect in each province and territory in Canada’ [34]. To exclude the possibility of identifying spurious associations between ticks and human cases acquired outside the province, any *I*. *scapularis* tick submissions or cases for which there was a history of travel outside the province 30 days prior to a tick being detected, or a case being diagnosed, were not included in the analyses.

The passive tick surveillance data comprise the number of single-tick submissions from each CSD where the tick was acquired. Our objective was to calibrate the relationship between the numbers of tick submissions in passive surveillance and the number of human Lyme disease cases. It is likely that in some CSDs with no reported Lyme disease cases, *I*. *scapularis* tick populations have recently become established (there can be a lag of several years between tick populations becoming established and there being significant Lyme disease risk; [13]), and being better able to identify such CSDs using passive surveillance data is an objective of this study. To reduce the possibility of false negative signals from the passive surveillance data, CSDs with tick submissions but no human cases were, therefore, removed from the analysis. CSDs with no tick submissions were also excluded because these may be situated outside the current geographic range of the tick (in which case human cases would be rare) [25], or they may not contain medical clinics participating in the passive surveillance program (in which human cases may well occur in the absence of submitted ticks).

In Ontario, Public Health Ontario discontinued passive tick surveillance in the health units of Kingston-Frontenac and Lennox & Addington; Leeds-Grenville and Lanark District; and Eastern Ontario in 2014 because the presence of tick populations and Lyme disease risk was well known in these locations [35]. Consequently, data from CSDs within these health units from 2014 onwards were not available for the analysis.

### Development of the risk indicator

We used the methodological of Ripoche et al. (2018) [26] as described briefly in the following

#### Modelling relationship between passive tick surveillance and Lyme disease human cases surveillance

Two models were built to assess the relationship between the outcome variable of human Lyme disease cases (confirmed and probable cases combined), and data from passive tick surveillance at the CSD level, conducted from 2009–2015. The numbers of cases and tick data from two successive years were combined into one datum for each CSD. In model 1, the explanatory variable was the cumulated number of all *I*. *scapularis* submissions (adults and nymphs) over two years contemporaneous with the Lyme disease case surveillance data. In model 2, the explanatory variable was the number of nymph submissions over the same two years. Given the small number of nymphs submitted through passive tick surveillance in Manitoba, the latter analysis was performed only on data from Ontario. In both models, the natural log of human population size was included as an explanatory variable because we expect the number of tick submissions and occurrence of Lyme disease cases to increase with the human population size [17, 25]. Model 1 and model 2 used a Generalized Estimating Equation (GEE) with an extended Poisson regression and a negative binomial response for human Lyme disease cases. The GEE procedure handles dependency of repeated measurements of CSDs over years and adjusts for overdispersion by robust standard errors [36]. The extended Poisson regression can account for over-dispersed count data of the outcome variable [37]. Potential interaction between population size and tick submissions was explored as well. Multicollinearity were assessed by calculating the variance inflation factor (VIF) among explanatory variables [38]. Model fit was assessed by graphical inspection of the residuals. Cook’s distance was computed to detect outliers. CSD’s with Cook’s distance > 1 that may influence the estimate of our statistical models were examined to determine whether they should be removed from the analysis [39]. When apparent outliers were detected, additional analyses were performed with and without the outlier CSDs to detect possible variation in the estimates obtained. When no difference in the estimates was found, the CSDs were kept in the models, otherwise they were dropped from analysis.

#### Assessment of the performance of the risk indicator to discriminate CSD with at least three reported human cases

As in Ripoche et al. (2018) [26], the capacity of the number of tick submissions from people (the predictor variable) per CSD cumulated over five-year periods from 2009 to 2015 was assessed for its capacity to discriminate between CSDs with ≥ 3 and those with < 3 human cases cumulated over the same time period. These five-year cumulated data were found previously to perform best at discriminating CSDs with different Lyme disease incidence likely because of interannual variability in tick submissions and Lyme disease case reporting. For simplicity, and given that tick submissions and reported human LD cases are coming from the same populations in each CSD, for this analysis the predictor was not adjusted for human population size.

We used Receiver Operating Characteristic (ROC) curves to assess the performance of the predictor variable [38]. An at-risk CSD was defined as one in which ≥ 3 locally-acquired Lyme disease cases had been reported within five years. To identify the optimal cut-off point value of the number of *I*. *scapularis* submissions, that minimises misclassification [40], sensitivity (Se) and specificity (Sp) curves were plotted [41]. The cut-off point value yielding the best combination of Se and Sp was considered the value at which the Se and Sp are equal [42].

The performance of the indicator was assessed by the positive predicted values (PPV = True positives / [true-positives + false-positives]) and negative predictive values (NPV = True-negatives / [true-negatives + false-negatives]) obtained using each optimal cut-off point. Finally, we investigated the ability of each optimal cut-off point to ‘forecast’ the incidence of human Lyme disease cases in the subsequent year. For this analysis, 2014–2015 cumulated *I*. *scapularis* tick submissions to forecast the numbers of Lyme disease cases in 2016. The hypothesis tested here is that if the number of submissions in a CSD reaches the cut-off point in two years rather than five, the number of human cases in year 3 may be three or more.

Statistical analyzes were performed with IBM SPSS Statistics version 24 (IBM, Chicago, IL, USA).

#### Mapping significant Lyme disease risk

Maps identifying CSDs with significant Lyme disease risk were created based on the optimal cut-off point of the cumulated five-year blacklegged tick submission data for Ontario and Manitoba in year 2015. CSDs with 1−2 human Lyme disease cases were also mapped. All maps were created using Environmental Systems Research Institute’s ArcGIS v10.5 software (Redlands, California).

## Results

### Data collected through passive tick surveillance and human Lyme disease surveillance programs

From 2009 through 2015 11,247 individuals submitted one *I*. *scapularis* through the passive surveillance program and ticks were reported to be acquired in 291 different CSDs in Ontario (Table 1). Of these blacklegged ticks, 10,590 were adults and 640 were nymphs, and the prevalence of *B*. *burgdorferi* infection was 17.6% (95% CI, 16.9–18.3) in adults and 12.7% in nymphs (95% CI, 10.0–15.9). During the same period, 414 individuals submitted one *I*. *scapularis* (adults = 377, nymphs = 37) acquired in 81 different CSDs in Manitoba. In total 22.3% (95% CI, 18.5–26.5) and 25.0% (95% CI, 12.1–42.2) of adults and nymphal blacklegged ticks were infected, respectively.

**Table 1:** Results of Lyme disease surveillance in humans and passive *I*. *scapularis* tick surveillance from 2009 through 2016 in Ontario and Manitoba.

		2009	2010	2011	2012	2013	2014	2015	2016
Ontario	*Lyme disease human case surveillance*								
	Total cases	37	38	85	92	184	144	323	287
	No. of CSDs with ≥ 1 case	18	18	31	37	54	45	74	85
	No. of CSDs with ≥ 3 cases over 5 years	0	0	0	0	21	23	28	23*
	*Passive* I. scapularis *surveillance*								
	No. of adult and nymphal ticks collected	590	677	1,778	2,064	2,701	1,911	1,526	n/a
	No. of CSD ≥ 1 single tick submission	70	92	139	151	178	191	205	n/a
	No. of nymphs collected	19	45	69	93	270	52	92	n/a
	No. of CSD ≥ 1 single nymph submission	9	21	25	35	62	28	50	n/a
Manitoba	*Lyme disease human case surveillance*								
	Total cases	0	4	7	12	19	28	22	33
	No. of CSDs with ≥ 1 case	0	2	4	3	9	17	12	19
	No. of CSDs with ≥ 3 cases over 5 years	0	0	0	0	4	5	8	2*
	*Passive* I. scapularis *surveillance*								
	No. of adult and nymphal ticks collected	16	37	42	56	89	84	90	n/a
	No. of CSD ≥ 1 single tick submission	8	24	23	29	37	39	41	n/a

* Includes only CSDs with ≥ 3 Lyme disease cases reported in 2016.

From 2009 through 2015, 903 Lyme disease cases were reported as having acquired infection in 137 different CSDs in Ontario and 92 cases were reported as having acquired infection in 28 different CSDs in Manitoba (Table 1). Except for Manitoba in 2015, there was a steady increase in the incidence of locally acquired cases, and in the number of CSDs where Lyme disease was acquired.

### Development of the risk indicator

#### Evaluation of the relationship between passive tick surveillance and human lyme disease surveillance

**Fig 1:**
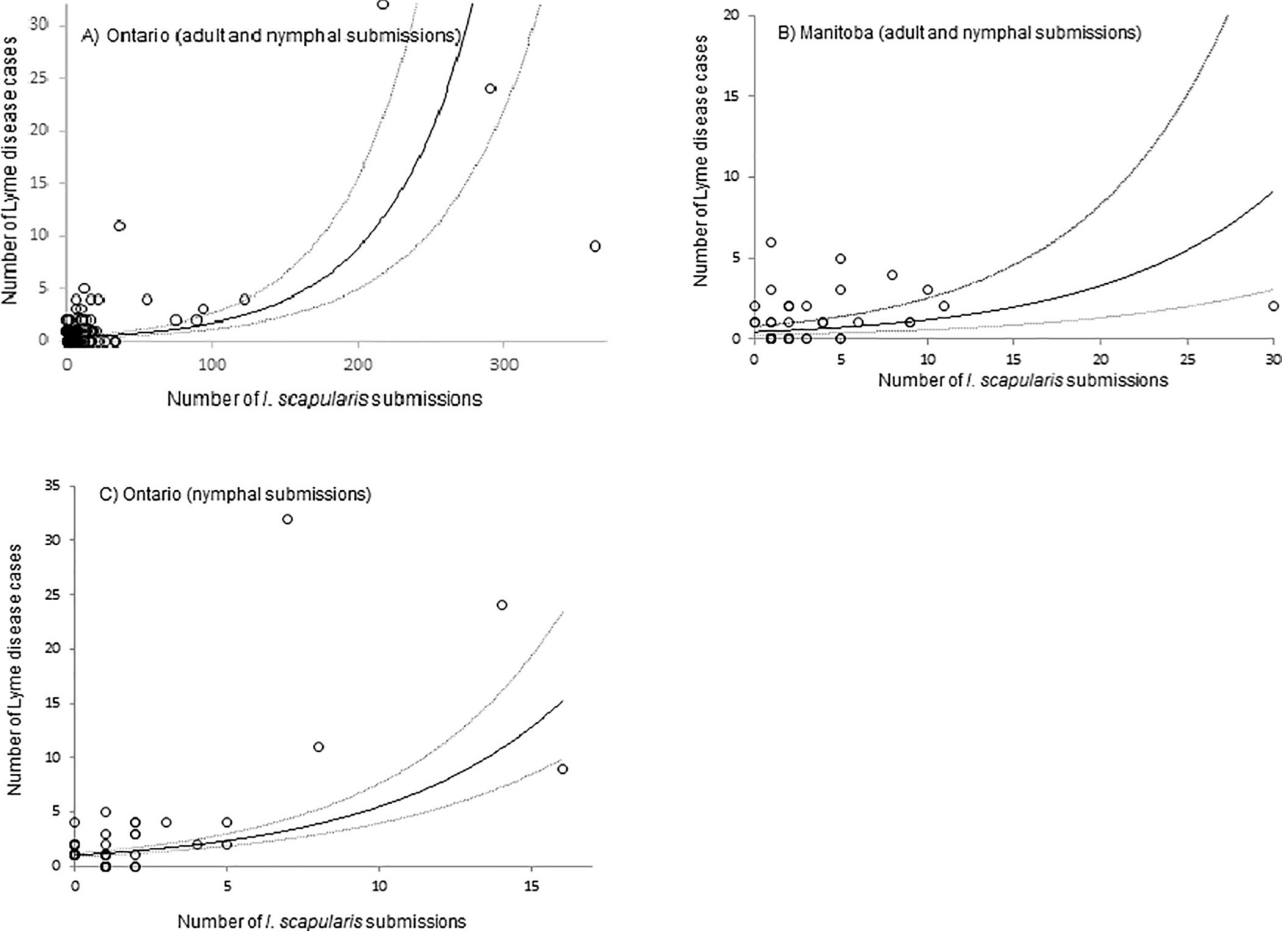
Negative binomial generalized estimating equation models of the relationship between the numbers of passive single *I*. *scapularis* tick submissions and reported Lyme disease cases per CSD, over 2 years. **Numbers of tick submissions for all *I*. *scapularis* (adults and nymphs) in Ontario and Manitoba are illustrated respectively in A and B, whereas the numbers of nymphal tick submissions for Ontario are shown in C. The numbers of reported Lyme disease cases per CSD are illustrated by the circles and the model prediction by the solid line (dashed lines show the 95% confidence limits for the fitted model).** Note that there are differences in the axis scales between the Figs.

Using Ontario data in Model 1, the number of tick submissions (adults and nymphs combined) and the population size were positively associated with the number of human Lyme disease cases (*P* < 0.001), (Table 2, Fig 1A). There was a negative interaction (*P* < 0.001) between tick submissions and population size (i.e. as human population size increases, the strength of the relationship between numbers of tick submissions and numbers of human cases gets smaller) and there was no significant collinearity between these variables (VIF < 3). Using Manitoba data in Model 1, the number of Lyme disease cases increased with the number of tick submissions (adults and nymphs combined) (*P* < 0.01), (Table 2, Fig 1B).

**Table 2:** Negative binomial generalized estimating equation models testing for the influence of the number of *I*. *scapularis* tick submissions on the occurrence of human Lyme disease cases; adult and nymphal *I*. *scapularis* submissions (Model 1) and nymphal *I*. *scapularis* submissions (Model 2) detected by the passive tick surveillance program in Ontario and Manitoba.

Model		No. of municipalities	Parameter	Estimate (β)	Standard errors (SE)	95% confidence limits	*P-value*
**Model 1**	Ontario	1,121	Intercept	-3.074	0.707	-4.459–1.689	0.000
			Adult and nymphal tick submissions	0.045	0.007	0.031 0.059	0.000
			Ln(CSDpop)	0.106	0.037	0.034 0.179	0.004
			Adult and nymphal tick submissions*Ln(CSDpop)	-0.001	0.000	-0.002–0.001	0.000
							
	Manitoba	280	Intercept	-0.855	0.346	-1.534–0.176	0.014
			Adult and nymphal tick submissions	0.102	0.031	0.041 0.164	0.001
							
**Model 2**	Ontario	470	Intercept	-0.002	0.084	-0.167 0.164	0.984
			Nymphal tick submissions	0.170	0.014	0.143 0.198	0.000

Using Ontario data in Model 2, the number of human Lyme disease cases increased significantly with the number of nymph submissions (*P* < 0.001), (Table 2, Fig 1C). The measure for influential data points using Cook’s distance detected three outliers in Ontario for all tick submissions in Model 1. However, the computation of the Model 1 without these influential CSDs did not change significantly the estimate and hence these CSDs were kept in the model.

#### Assessment of the performances of the risk indicators

**Fig 2:**
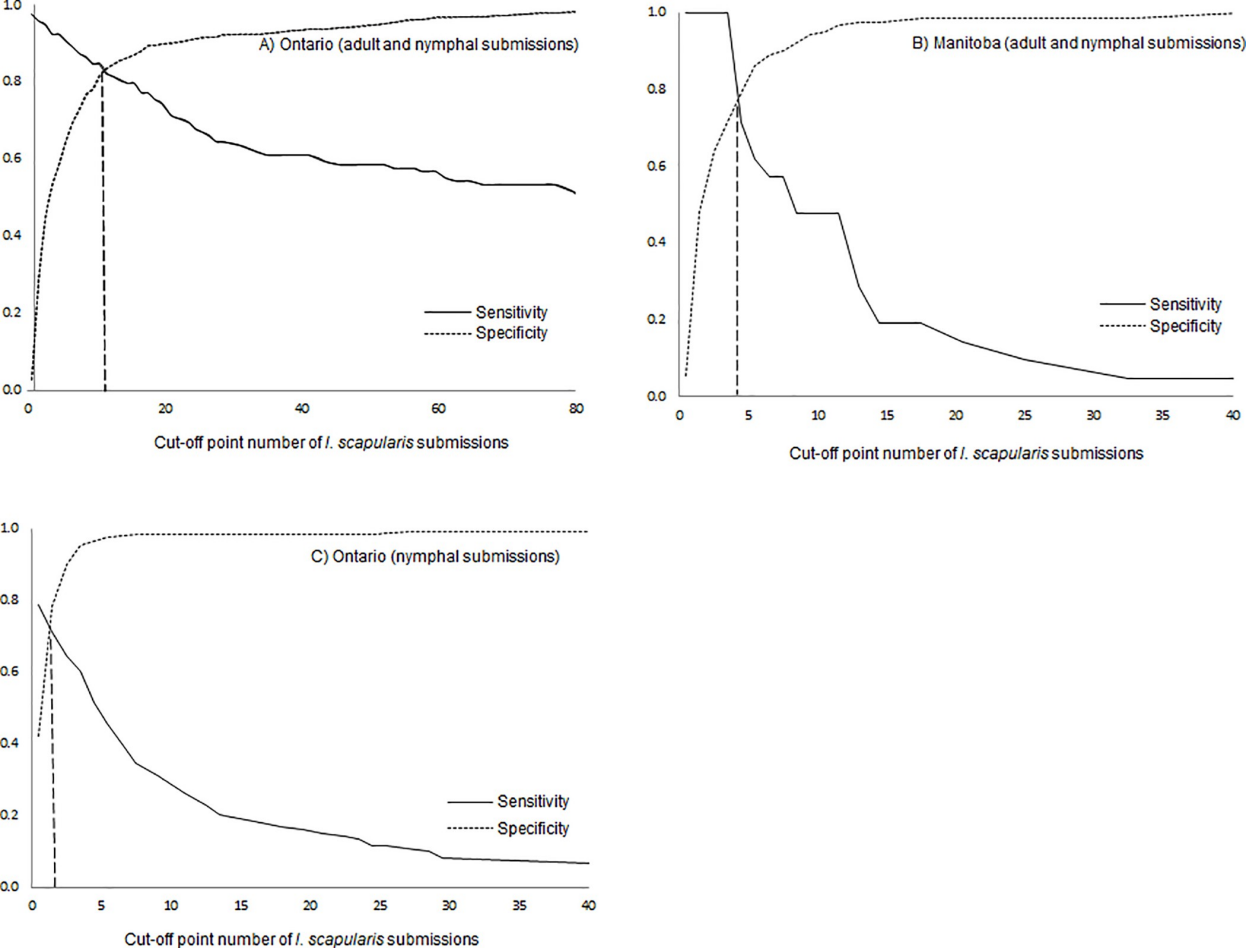
Sensitivity and specificity curves of the range of cut-off point number of *I*. *scapularis* (adults and nymphs) submissions in Ontario and Manitoba (A and B) and nymphal *I*. *scapularis* submissions in Ontario (C). The straight dashed line corresponds to the intersection between Se and Sp curves, the optimal cut-off point which maximize the Se and Sp over a range of cut-off points of the number of tick submissions. A− C illustrate the optimal cut-off points of cumulated 12, 5 and 2 tick submissions over five-year period to discriminate CSDs with ≥ 3 human Lyme disease cases.

For Ontario, the total numbers of blacklegged tick submissions was moderately accurate (using the definition of Greiner et al. 2000) at predicting the occurrence of CSDs with ≥ 3 Lyme disease cases (AUC = 0.89) and a cut-off point of 12 *I*. *scapularis* submissions was associated with the highest performance (Se = 0.82, Sp = 0.84) (Table 3, Fig 2A). Validation of this optimal cut-off point to the current epidemiologic situation showed that 84% of the CSDs were correctly classified when compared to CSDs where Lyme disease cases were reported (Table 3).

**Table 3:** The performances of the optimal risk indicators and their validation under the current epidemiologic Lyme disease situation.

		Municipalities		ROC Curve			Indicator performances			Test validation			
**Province**	Parameter	Total	Prevalence	AUC	AUC CI 95%	*P-value*	Cut-off point	Se	Sp	% CSDs	PPV	NPV
**Model 1. Adult and nymphal *I*. *scapularis* tick submissions from passive surveillance as predictor of human Lyme disease risk-area**												
**Ontario**	Adult and nymphal tick submissions	822	0.14	0.89	0.85 − 0.92	0.000	12*	0.82	0.84	0.84	0.51	0.97
**Manitoba**	Adult and nymphal tick submissions	223	0.09	0.89	0.84 − 0.94	0.000	5*	0.71	0.79	0.79	0.30	0.97
**Model 2. Nymphal *I*. *scapularis* submissions from passive surveillance as predictor of human Lyme disease risk-area**												
**Ontario**	Nymphal tick submissions	436	0.27	0.78	0.72 − 0.84	0.000	2*	0.71	0.78	0.79	0.64	0.86

*: Optimal cut-off point number of *I*. *scapularis* tick submissions maximizing sensitivity [Se] and specificity [Sp] and where Se and Sp are approximatelly equal.

Prevalence: municipalities (equivalent to census subdivisions) where ≥ 3 human Lyme disease cases are reported to be acquired in, among tested municipalities. AUC: Area Under Curve. ROC: Receiver Operating Characteristics. % CSDs: proportion of census subdivisions correctly classified. PPV: Positive Predictive Value. NPV: Negative Predictive Value. Test validation: validation of the cut-off point number of *I*. *scapularis* submissions under current epidemiologic situation.

For Manitoba, the total numbers of Blacklegged tick submissions was also moderately accurate at predicting the occurrence of CSDs with ≥ 3 Lyme disease cases (AUC = 0.89) and a cut-off point of five submissions was associated with the highest performance (Se = 0.71, Sp = 0.79) (Table 3, Fig 2B). After validation of the cut-off point to the current epidemiologic situation, 79% of the CSDs were correctly classified when compared to CSDs where Lyme disease cases where reported (Table 3).

Also for Ontario, two *I*. *scapularis* nymph submissions, indicate with moderate performances (AUC = 0.78, Se = 0.71, Sp = 0.78) the CSDs where the risk from Lyme disease is occurring (Table 3, Fig 2C). After validation of the cut-off point to the current epidemiologic situation, 79% of the CSDs were correctly classified when compared to CSDs where Lyme disease cases were reported (Table 3).

Using the optimal cut-off point for each province, cumulative numbers of tick submissions from 2014 through 2015 correctly categorized CSDs as high risk (≥ 3 cases) and moderate risk (< 3 cases) in 83% and 85% of cases (respectively for Ontario and Manitoba) using Lyme disease case data from 2016. Also, in Ontario, a cut-off point of two nymphs correctly categorized the risk level of 74.5% of the CSDs obtained using 2016 Lyme disease case data.

#### Mapping significant Lyme disease risk

**Fig 3:**
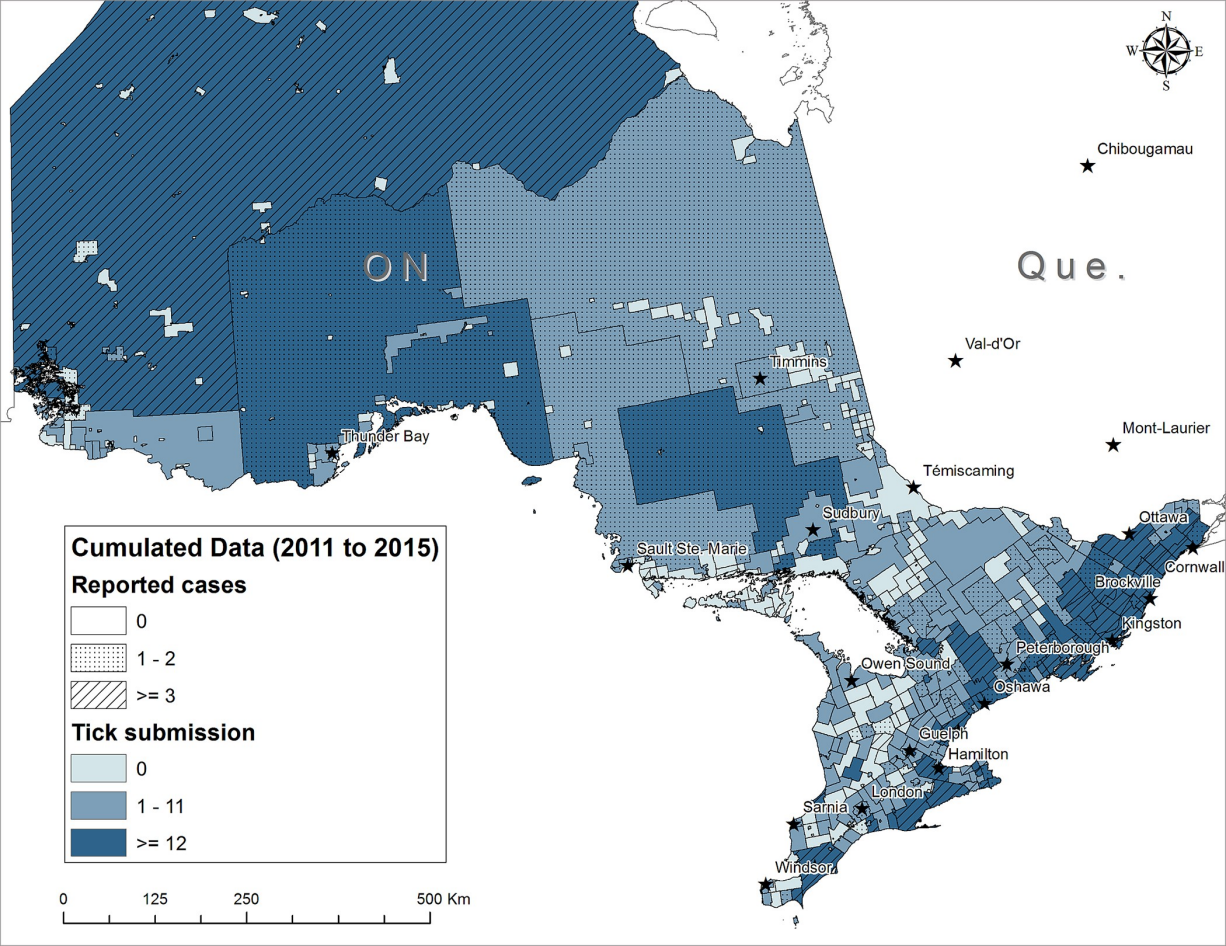
Geographic distribution of passive *I*. *scapularis* tick submissions (adults and nymphs) and total number of reported human Lyme disease cases in CSDs in Ontario from 2011 to 2015. Dotted CSDs are areas with emerging risk (1–2 human Lyme disease cases). Hatched CSDs are endemic areas where ≥ 3 Lyme disease cases were acquired. Light blue CSDs are areas where the number of adult and nymphal is < 12, the optimal cut-off point as indicator of the human Lyme disease risk. Dark blue CSDs are areas where the number of *I*. *scapularis* submissions is ≥ 12.

**Fig 4:**
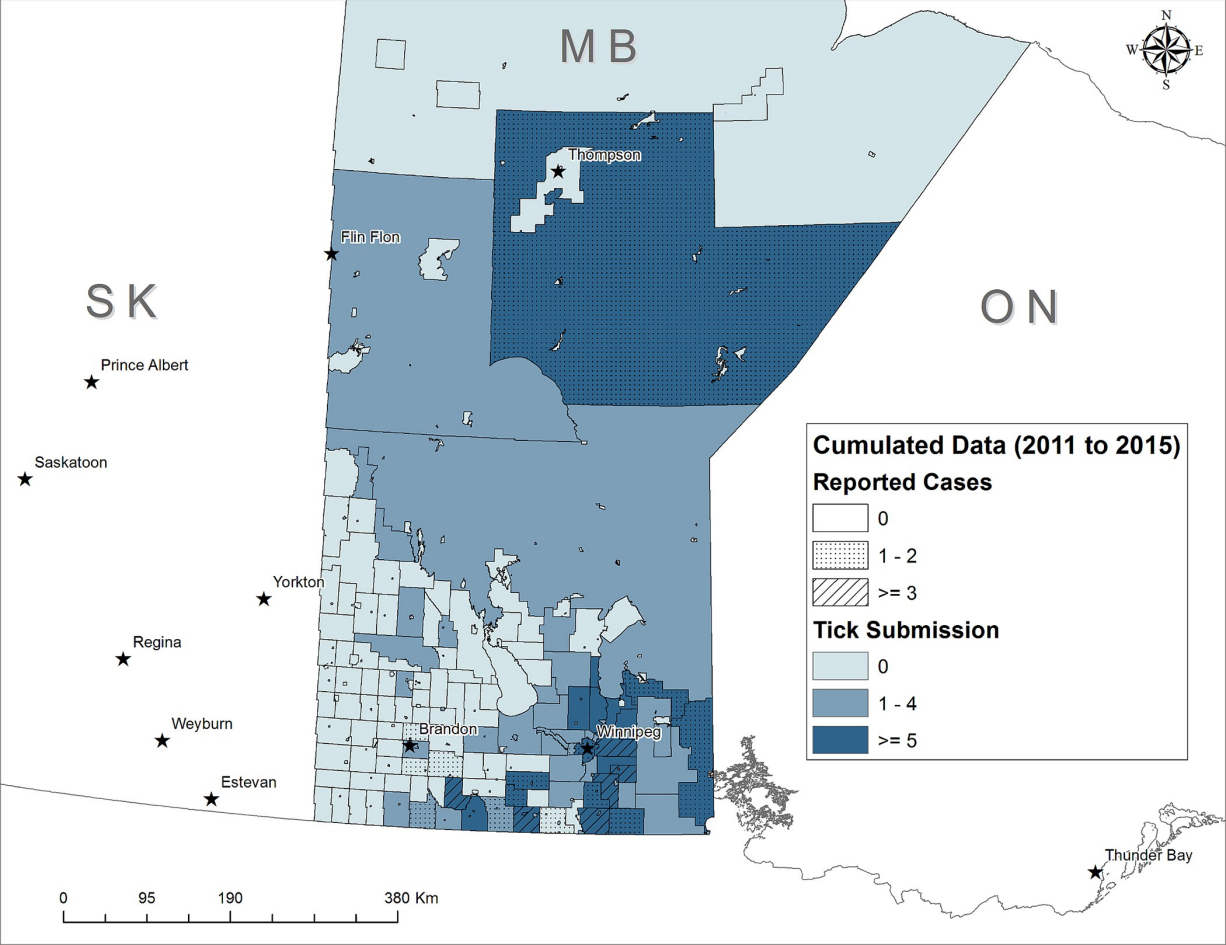
Geographic distribution of passive *I*. *scapularis* tick submissions (adults and nymphs) and total number of human Lyme disease cases in CSDs in Manitoba from 2011 to 2015. Dotted CSD are areas with emerging risk (1–2 human Lyme disease cases). Hatched CSD are endemic areas where ≥ 3 Lyme disease cases were acquired. Light blue CSDs are areas where the number of adults and nymphs is < 5, the optimal cut-off point as a predictor of the human Lyme disease risk. Dark blue CSDs are areas where the number of ticks is ≥ 5.

Maps showing significant Lyme disease risk according to numbers of tick submissions and numbers of Lyme disease cases are shown in Figs 3 and 4 for Ontario and Manitoba, respectively. In each map, the risk is characterized according to the human Lyme disease surveillance cases (as ‘moderate incidence’ if CSDs had 1−2 human Lyme disease cases, and ‘high incidence’ whereas CSDs with ≥ 3 cases overlaying the risk according to the optimal cut-off point of each province).

## Discussion

Our study shows that passive blacklegged tick surveillance data is specific and sensitive for detecting CSDs in Ontario and Manitoba where human cases of Lyme disease are most expected. The present work expands on our previous development of an acarological indicator of areas where the risk of Lyme disease transmission in Québec by providing province-specific indicators for Ontario and Manitoba [26].

We found a strong positive relationship between blacklegged tick submissions in passive tick surveillance and reported human Lyme disease cases in Ontario and Manitoba. However, in contrast with the previous study in Quebec, occurrence of Lyme disease cases increased with the size of the human population as well as with the numbers of tick submissions. We speculate that in Quebec more CSDs with higher human population density are found at the edge of the advancing front of *I*. *scapularis* tick invasion, where ticks would be found and submitted, but human case incidence may be low due to low *B*. *burgdorferi* infection prevalence in the newly established tick populations [13]. This would also mean that in Ontario and Manitoba, more CSDs with higher human population density are found in locations with more mature tick populations with greater infection prevalence. Certainly infection prevalence in ticks collected in Manitoba and Ontario (this study) was greater than that in ticks collected in Quebec (14.3% [16]).

The accuracy to discriminate CSDs with high incidence of Lyme disease (≥ 3 human Lyme disease cases) versus moderate incidence (< 3 cases) was moderate but acceptable (AUC approaching 0.9) when using total blacklegged tick submissions in the two provinces. In Ontario, a cut-off point of 12 tick submissions over the previous five years discriminated with high Se and Sp, those municipalities with three cases or greater versus fewer than three cases during the same period. With this optimal cut-off point, 16% of CSDs were misclassified, with most being false positives, suggesting that the indicator has a tendency to over predict CSDs with human risk which is similar to the Québec indicator [26]. As reported previously, the issue in model fitting may be due to factors such as: i) failure to detect or under-reporting of Lyme disease cases [43]; ii) adoption of preventive measures that prevent Lyme disease transmission [44]; and iii) low prevalence of infectious blacklegged ticks in emerging areas [13]. Further studies are needed to explore these and factors such as environmental variability that may impact where and when relationships between numbers of tick submissions and human cases may vary [25].

The ability of the indicator to predict in advance the human Lyme disease risk based, on the cumulated two years’ tick submissions for 2014 − 2015, showed that the cut-off point of 12 tick submissions discriminated correctly 83% (with 9.7% being false positive CSDs) of the CSDs with ≥ 3 human Lyme disease cases versus < 3 cases in the subsequent year.

Densities of infected *I*. *scapularis* nymphs in surveillance are more predictive of Lyme disease cases than adult ticks where tick populations have been established [45]. Because of their small size, nymphs are harder to detect and thus can remain attached longer on their hosts increasing the chance of transmission of *B*. *burgdorferi* [46]. We found positive relationship between nymph submissions from passive surveillance and human Lyme disease cases in Ontario. However, in this study and in the study conducted in Québec [26], the accuracy of nymph submissions alone to discriminate municipalities with high Lyme disease incidence was moderate and lower than when including adult tick submissions. Most likely this suggests that, because of their small size, nymphs go undetected by the general human population and consequently are less likely to be captured by the surveillance system. However, this finding may in part be due to the cessation of passive tick surveillance from three health units along the north shore of Lake Ontario/St. Lawrence River where most of nymphs were submitted from 2008 through 2012 [47]. The usefulness of passive blacklegged tick surveillance data to estimate Lyme disease risk areas has been reported in several studies in North America [16–18, 25, 26, 48–51] and in Europe [52]. We demonstrate that passive tick surveillance data can predict regions where Lyme disease risk areas are emerging. The ongoing range expansion of *I*. *scapularis* in Canada is expected to mean that 80% of the population will live in areas where there may be risk from Lyme borreliosis [53]. The specific acarological risk indicators developed for Ontario and Manitoba enhance our knowledge of where tick populations and *B*. *burgdorferi* transmission cycles are established now. Our risk indicator contributes to risk map development, contributing to more targeted preventive or control measures, as well as supporting the early clinical diagnosis of Lyme disease and deployment of antimicrobial prophylaxis [54]. The areas of risk identified by the analysis here (Figs 3 and 4) are consistent with known risk areas identified by active surveillance (see https://www.publichealthontario.ca/en/eRepository/Lyme_disease_risk_areas_map.pdf and https://www.gov.mb.ca/health/publichealth/cdc/tickborne/surveillance.html respectively for Ontario and Manitoba). Clearly though, full confirmation of the existence of *I*. *scapularis* populations in a CSD identified as likely holding *I*. *scapularis* populations by passive surveillance, and identifying specifically what woodlands in a CSD hold populations of the tick would require active field surveillance.

We found it interesting that the acarological signal in Québec and Ontario was very similar (11 and 12 tick submission optimal cut-off points, respectively) while in Manitoba the indicator was much lower (five tick submission optimal cut-off point), suggesting a higher risk of Lyme disease acquisition in Manitoba. This finding may reflect differences in the ecology of Lyme disease in these regions, including possible differences in tick seasonal activity patterns that may affect both tick infection prevalence [55, 56], and geographic variation in occurrence of *B*. *burgdorferi* strains [57], which have different capacity to cause disease [58, 59]. The infection prevalence of ticks collected in Manitoba was greater than in Ontario. While this may reflect different stages of tick population establishment in these regions [13], this is consistent with differences observed between ticks found in the upper Midwest and northeastern US [60]. This finding could also reflect some inter-provincial variations in passive surveillance and rates with which ticks biting people are submitted by the public.

Previous studies on passive tick surveillance data have identified a lack of specificity, associated with collection of adventitious ticks seeded by migratory birds [18]. When a high proportion of ticks are collected from dogs and other pets, which are very efficient at acquiring ticks from the environment [24]. However, in our data of ticks submitted from humans, adventitious ticks likely represent a minority of all ticks making up an established tick population.

## Conclusion

Given the limited geographical coverage of active field surveillance and the possible gaps from human Lyme disease surveillance, we developed a province-specific acarological indicator that provide information for risk maps complementary to that provided by active tick surveillance and human Lyme disease surveillance. The risk indicator provides the particularly relevant information as it is based on a risk-measure of human/tick encounter. Despite the limitations of passive tick surveillance systems, we concluded that when analyzed rigorously, ticks collected from people can be used as a useful tool for public health authorities to identify Lyme disease risk areas. This type of methodological approach warrants consideration in other regions of North America where the range of ticks and tick-borne pathogens are expanding.
